# Typhoid Fever as It Appeared in the Vicinity of Heltonsville during the Winters of 1846-’7-’8

**Published:** 1849-03

**Authors:** M. H. Fee

**Affiliations:** Heltonsville, Indiana


### WESTERN JOURNAL

OF

### MEDICINE AND SURGERY.

MARCH, 1849.

Art. I.—*Typhoid Fever as it appeared in the vicinity of Heltonsville during the winters of* 1846–'7–'8. By Dr. M. H. Fee, of Heltonsville, Indiana.

The winters above indicated were characterized by great and frequent thermometrical, barometrical, and electrical changes. Torrents of rain fell frequently, and the weather was generally wet, followed, for a few days, by great depressions of temperature. It is not at all strange, I think, that amid so much wet, our diseases were generally of the asthenic type. Typhoid fever prevailed to a considerable extent, but was confined almost entirely to our streams and alluvions. During the prevalence of this fever in the lowlands, catarrhal fever visited the highlands. Chronic cases of ague were constantly relapsing, with tendency to dropsical effusions.

Cancrum oris prevailed almost universally; but was much worse in the low damp air of the streams. In short, there was unmistakeable evidence of diminished vital resistance. We had three varieties of typhoid which I shall designate typhoid with *Peyerian complication;* typhoid with *pneumonic complication;* and typhoid with *congestion of the portal circulation.* In the remarks which follow I shall keep this division in view. For the sake of perspicuity I shall speak 1st, of the initiatory symptoms of each variety; 2d, of the symptoms after complete development; 3d and last, of the treatment which proved most successful.

1st. In the Peyerian form, I have seen patients sometimes for ten days before active development, with melancholy appearance, eyes sunken and inanimate; icterode cast of the skin and conjunctiva; slight *malaise;* confusion of ideas, and intellectual dullness; sudden and transient flushing of the face; sense of stricture across the forehead; slight vertigo toward evening; heavy sleep, with occasional muttering or groaning; tongue coated with a white fur, the papillæ showing enlarged and intensely red through the coat; a sense of weight and dryness in the bowels generally, with constipation. The urine is mostly scanty, thick, and red; aching in the loins; the hands and feet constantly icy cold, and not sensible to the impression of capsicum. I will observe here, that this enlargement of the papillæ of the tongue is an infallible diagnostic, in adults, of inflammation of Peyer's glands.

2d. *Of the initiatory symptoms of the pneumonic variety.*—In this variety we have the usual symptoms of catarrhal fever, viz: flushed face; injected and watery eyes; acute pain in the forehead; hyper-schneiderian secretion; soreness of the fauces; thickness of voice; thoracic stricture; cough, with transient pains in one or both lungs; great sensitiveness to the impression of cold; slight febrile exacerbations towards evening; stupor of the intellectual faculties; violent cough during the early part of the night; a disposition to sleep. The bowels are rarely prominently deranged. These are some of the most prominent initial symptoms.

3d. *Of the initial symptoms of the portal variety.*—In this variety, in addition to the usual symptoms of the other forms of typhoid, indicating an asthenic tendency, we have usually a fulness and pain of the right hypochondriac region, with frequent and easy discharges of a white thin foamy fecal matter, destitute of bile; great nervousness, with weakness of the joints; syncopic tendency upon the slightest exertion; aching of the back, etc., etc., etc.

1st. *Of the symptoms after complete development of the Peyerian variety.*—The papillæ of the tongue much enlarged, showing red through a brownish white fur; edges and tip of tongue dry and red; stupor, with somnolency; disinclination to answer questions; taciturnity, answering as much as possible by monosyllables; pulse about 120, sometimes 140 per minute; gurgling sound in the bowels when liquids are swallowed; tenderness just below the umbilicus; vivid, circumscribed flushes upon the face and neck on the slightest exertion; constipated bowels; urine scanty and red, sometimes copious and pale; nervous twitchings. As the disease advances, muttering delirium appears; meteorism; floccitacio; roseola on the neck and breast; papulæ on the neck, which is sallow, enlarged, and in a few days filled with a limpid serum; the lips and teeth covered with sordes; the tongue covered with a dark brown and cracked coat, with patches clean, florid, dry, and covered apparently with a delicate membrane; retention of urine, or stillicidium. As the disease advances the tongue becomes pitted, which pits are filled with what appears to be pus. This is a certain indication of ulceration of Peyer's and Brunner's glands. About this time a gentle, sometimes profuse, diarrhea comes on, at which time we may frequently trace in the feces globules of matter as large as "bird shot," evidently from the ulcerated glands, etc., etc.

2d. *Symptoms after complete development of the pneumonic variety.*—Harrassing cough; pain in one, sometimes both lungs, shooting up into the shoulders; inability to lie on the affected side; stridulous respiratory murmur in the affected lung; body inclined towards the affected side; quiescent state of that side of the chest; diaphragmatic respiration; coughing up of a greenish viscid mucus, sometimes tinged with blood; rapid panting breathing; heavy expression of the eyes; tongue covered with a whitish yellow fur; anorexia, with pyrexia; bowels not difficult to move; pulse full, frequent, and moderately hard, though of easy compression; distinct chill, or at least a cold stage, every morning about 9 o'clock, followed by a moderate degree of fever till the middle of the evening; considerable dyspnea during the febrile excitement; considerable desire for cool drinks, with acute pain in the head, and circumscribed flush on the centre of one, sometimes both cheeks, etc.

3d. *Symptoms after complete development of the portal variety.*—If the congestion is of some considerable antiquity, the stomach will be found quite irritable, rejecting all liquids and solids almost as soon as they are swallowed; but if of more recent date, the stomach does not sympathize strongly. We have incessant purging of watery, colorless, or white rice-water discharges, without a trace of bile; no thirst of cosequence; the surface blue and shrunken, with *cutis anserina.* The patient seems almost a third less than formerly; great anxiety; grunting expiration; constant syncopic tendency; hands and feet icy cold, and the skin on them drawn into wrinkles, of a dirty white appearance, as we often see it in cholera. There are frequently spasms of great severity during this collapsed stage, as I am in the habit of calling it. Last winter, I had a patient who had forty-eight alvine dejections, of the above character, in twenty-four hours, during the congestive stage. The pulse is small, almost imperceptible, and rapid, sometimes amounting to 150 in the minute. The tongue is pale and bloodless, covered with a long pale fur; upon protruding the tongue it is found broad, flabby, and tremulous.

1st. *Treatment of the Peyerian variety.*—In this complication I never resort to bloodletting. Indeed, when we consider that local glandular inflammation is but a secondary feature, and that a disease of asthenic tendency is the subject of our treatment, we may well pause and inquire, is not general bloodletting, to say the least of it, a remedy of very doubtful utility? There is already diminished vital force; the all important question then is, is general bloodletting likely to increase the vital force? I think not. The great desideratum is to change the morbid action of the system, without diminishing its resources. When called to a patient with this complication, I am in the habit of applying wet cups to the abdomen, and in this way drawing from two to four ounces of blood, and afterwards of covering the bowels with a large emollient poultice. If this treatment has been promptly and energetically adopted, it will be found that the papillæ of the tongue have diminished much in size, and lost, in some good degree, their vivid redness; I then give from thirty to forty grains of calomel, combined with from a half to three-quarters of a grain of morphia, to adults, which I let remain from sixteen to twenty-four hours, then move off with Seidlitz powder, and laxative and stimulant enemata. When I have evidence of a healthy action of the liver, I give no more mercurials, nor active purgatives, but use Seidlitz powders exclusively. For the *calor mordax* I sponge the body with warm water, and administer spirits of nitre and lemon juice every two hours. During the quieting effect of the calomel with the morphine, the tongue frequently becomes partially clean; but when the influence of the morphine has passed off, and the irritant effects of the calomel prevail, it is for a time often covered with a red, dry, and fine membrane, such as is seen thrown out over a blistered surface when about to commence the reparation of the cutis. But so soon as the liver has poured out a large quantity of vitiated bile, and this has passed off, we find the tongue suddenly become moist and much less florid, and the pulse reduced in frequency twenty to forty beats. Under the subsequent use of Seidlitz powder, or other mild laxatives, the tongue gradually loses its redness and the papillæ their size, until the normal condition of all the functions of the system is reëstablished. In this complication quinine canno be used in any dose, without positive injury, until after the local inflammation has terminated in resolution. If, after this happens, there be still daily exacerbations, quinine may be advantageously administered. If given before this time, the tongue becomes dry, and the bowels painful, without the slightest good impression being made on the succeeding paroxysm. If, despite my endeavors, the Peyerian glands go on to suppuration, known by pits of whitish yellow matter appearing on the tongue, I have been in the habit of meeting it by blistering the abdomen, and exhibiting nitrous, or sulphurous acid; but with the change my views have lately undergone, I should meet it with grain doses of nitrate of silver, every three hours, till the pits throw off the matter and assume a florid appearance, after which I should exhibit balsam copaiva or turpentine. Blisters, I think, only serve to retard the process of cicatrization of the glands by diverting to the surface, blood whose presence in the interior is all important. It would certainly be bad policy in an indolent ulcer of the leg to draw a blister on the thigh.

2d. *Treatment of the pneumonic variety.*—This variety, according to my experience, possesses more of the sthenic character than the one just treated of. The fever is more external, more completely developed and the pulse is of greater volume and hardness, than in either of the other varieties. If the pain in the chest be severe, and the cough violent, I premise a general bleeding, sufficient to make a decided impression on the pulse. If, after the bleeding, there be still considerable pain, I apply a few wet cups over its seat, followed by a warm emollient poultice. After proper depletory measures, I give thirty grains of calomel with half a grain of morphine, followed in twelve hours by half an ounce of castor oil. This generally serves to quiet the nervous irritability, and partially restore the secretions. For restoring the bronchial secretions I exhibit, every two hours, ½ʒf. spirits nitre, ʒss.f. syrupus scillæ, and ⅛gr. tart. antimony, until well digested pus makes its appearance in the sputa. This course, aided by blisters or powerful rubefacients upon the chest, generally reëstablishes the suspended secretion in three or four days. Mucilaginous drinks are freely used in the mean time. During the whole course I exhibit, at bed time each night, five grains blue mass, for the purpose of maintaining the biliary secretion. If the bronchial secretion becomes excessive, and the pulse small and rather weak, I give a tablespoonful, every three hours, of the following mixture:

℞. Infus. Seneg. ℥viii.

Aq. ammoniæ ʒi.

Syrup. scillæ ℥ss.

If, after the secretions have been pretty well established, there be periodic exacerbations of fever, I give quinine with decided advantage.

3d. *Treatment of the portal variety.*—In this variety, the first indication to be fulfilled is to equalize the circulation. The best way I have found to do this, is to rub the back and limbs freely with a stiff brush, as long as the patient will endure it. After this, apply to the parts just rubbed a flannel cloth, imbued moderately with very hot spirits of turpentine. During this operation let some one pour boiling water on some good wine or brandy, and let the patient drink it; this will aid materially in bringing about reaction. At the time of reaction, and for some time after, we are to watch closely that local visceral inflammation of some kind be not developed. As a general rule, however, the reaction is not violent, and we rarely have any very serious lesions. As soon as the dangers usually attending reaction are past, I turn my attention to the correction of the vitiated, or to the reëstablishment of the suspended, secretions. The secretions most implicated are those of the liver and skin; nothing suits the former better than mercury, combined with morphine; indeed, without some form of opium the calomel would pass rapidly through the bowels without effecting the secretion of bile. Thirty grains of calomel with one of morphine will generally arrest the diarrhea and keep the bowels quiet for twelve or sixteen hours—at the expiration of which time it may be moved off with castor oil. Blue mass I give occasionally to maintain the impression made by the calomel on the liver. When the bowels need moving, it should be done with castor oil, guarded with paregoric. For restoring the secretion of the skin I use nothing but spirits of nitre, aided by drinks of tea of M. pulegium, and warm bricks wrapped in wet cloths laid in bed near the patient. If diurnal febrile excitement continues after the secretions have been attended to, I use quinine to arrest them.

With this treatment in the years above specified, I have lost but 5 per cent. of all the cases of this fever treated in a scope of country of about ten miles square, abundant in streams and bottoms.

January, 1849.

